# Characterization of the complete mitochondrial genome of the lung fluke, *Paragonimus heterotremus*

**DOI:** 10.1080/23802359.2018.1462119

**Published:** 2018-05-11

**Authors:** Lei Qian, Ping Zhou, Weilin Li, Huaiping Wang, Tianjie Miao, Longgang Hu

**Affiliations:** a Department of medicine, Qingdao University, Shandong, Qingdao, China;; b The Women and Children’s Hospital of Qingdao, Shandong, Qingdao, China;; c The Affiliated Hospital of Medical College Qingdao University, Shandong, Qingdao, China

**Keywords:** *Paragonimus heterotremus*, mitochondrial genome, assembly, phylogeny

## Abstract

In this study, the complete mitochondrial genome of human lung fluke, *Paragonimus heterotremus,* was recovered through Illumina sequencing data. This complete mitochondrial genome of *P. heterotremus* is 13,927 bp in length and has a base composition of A (16.6%), T (41.8%), C (13.%), G (28.4%), demonstrating an obvious bias of high AT content (58.4%). The mitochondrial genome contains a typically conserved structure, encoding 12 protein-coding genes (PCGs), 22 transfer RNA genes (tRNA), 2 ribosomal RNA genes (12S rRNA and 16S rRNA) and a control region (D-loop region). All PCGs were located on the H-strand. ND4 gene and ND4L gene were overlapped by 39 bp. The nucleotide sequence of 12 PCGs of *P. heterotremus* and other 10 parasite species were used for phylogenetic analysis. The result indicated *P. heterotremus* a relative close relationship with species *Paragonimus westermani* (AF219379.2).

*Paragonimus heterotremus* is mainly distributed in Asia; China, Laos, Cambodia, and Thailand and can cause Paragonimiasis in human and other crab-eating mammals. Until now, the organelle genome information of *P. heterotremus* is still limited. In this study, the complete mitochondrial genome of *P. heterotremus* was recovered through Illumina Hiseq2500 sequencing. This complete mitochondrial genome can be subsequently used for clinical diagnosis and provide valuable insight into phylogeny relationship among *Paragonimus* species.

The eggs of *P. heterotremus* was collected from sputum of patients in Zhuang region, Guangxi Province, China (22°48′48.17″ N, 108°19′15.61″E). Adult worms were obtained by feeding dogs with metacercariae. Genomic DNA was extracted from adult worms using the commercial QiaAmp DNA extraction kit and DNeasy Tissue kit (supplied by Qiagen) according to the manufacturer's instructions. The isolated DNA was stored at −20 °C in the functional lab of Institute for Translation Medicine in Qingdao University. The partial genomic DNA was then subjected to standard Hiseq 2000 library construction. A total of 40 Gb reads were obtained with average length of 100 bp. After quality filtration, the clean reads were assembled by SPAdes 3.6.1 (Bankevich et al. 2012) based on default settings. We used another mitochondrial genome of *Paragonimus westermani* (AF219379.2) as a reference sequence to align the contigs and identify gaps. To fill the gap, Price (Ruby et al. 2013) and MITObim version 1.8 (Hahn et al. 2013) were applied and Bandage (Wick et al. 2015) was used to identify the circular topology. The complete sequence was primarily annotated by ORF prediction in Unipro UGENE (Okonechnikov et al. 2012) combined with manual correction. All tRNAs were confirmed using the tRNAscan-SE search server (Lowe and Eddy 1997). Other protein coding genes were verified by BLAST search on the NCBI website (http://blast.ncbi.nlm.nih.gov/), and manual correction for start and stop codons were conducted. The circular mitochondrial genome map was drawn using OrganellarGenomeDRAW (Lohse et al. 2007). This complete mitochondrial genome sequence together with gene annotations were submitted to GenBank under the accession numbers of MH059809.

The complete mitochondrial genome of *P. heterotremus* was 13,927 bp in length and has a base composition of A (16.6%), T (41.8%), C (13.%), G (28.4%), demonstrating an obvious bias of high AT content (58.4%). The mitochondrial genome contains a typically conserved structure, encoding 12 protein-coding genes (PCGs), 22 transfer RNA genes (tRNA), 2 ribosomal RNA genes (12S rRNA and 16S rRNA), and a control region (D-loop region). All PCGs were located on the H-strand. ND4 gene and ND4L gene were overlapped by 39 bp.

Phylogenetic analysis was constructed by applying 12 mitochondrial protein coding genes with other 10 closely related taxa. The whole genome alignment was constructed by HomBlocks (Bi et al. 2018) and verified by MAFFT (Katoh and Standley 2013). Finally, conserved regions were picked out by Gblocks 0.91b (Castresana 2002) to construct concatenated nucleotide sequences. Phylogenetic tree constructed using RAxML version 8.1.12 (Staamtakis 2014) and Mrbayes (Ronquist et al. 2012) was shown in Figure 1. The relationships among the 11 taxa were fully resolved with 100% values. *Paragonimus heterotremus* was clustered into the group of genus *Paragonimus* and exhibited a relative close genetic distance with *Paragonimus westermani* (AF219379.2).

**Figure 1.**
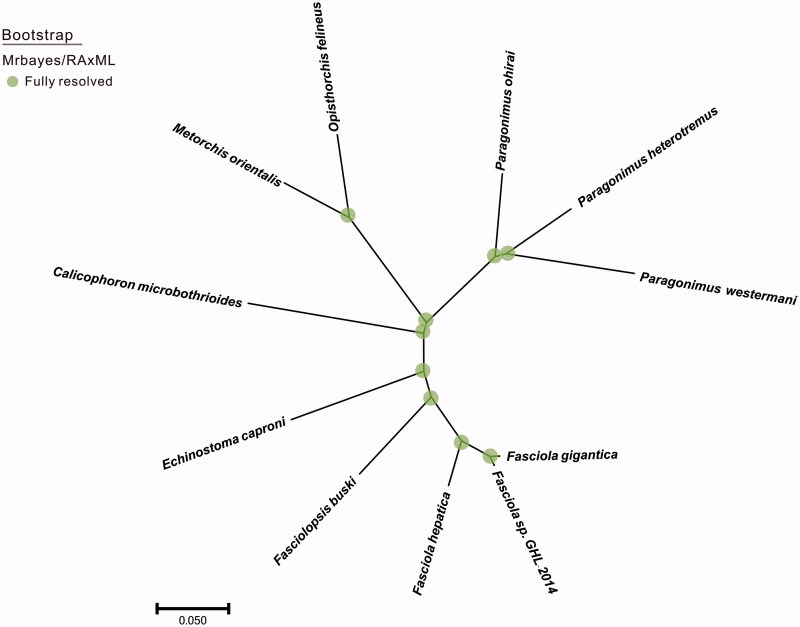
The phylogenetic tree of the 11 species from parasite species was constructed based on complete mitochondrial genome data. The analyzed species and corresponding Genebank accession numbers are as follows: *Paragonimus westermani* (AF219379.2), *Paragonimus ohirai* (KX765277.1), *Opisthorchis felineus* (EU921260.2), *Metorchis orientalis* (KT239342.1), *Calicophoron microbothrioides* (KR337555.1), *Echinostoma caproni* (AP017706.1), *Fasciolopsis buski* (KX169163.1), *Fasciola hepatica* (AF216697.1), *Fasciola* sp. GHL-2014 (KF543343.1), *Fasciola gigantica* (KF543342.1).

## References

[CIT0001] Bankevich A, Nurk S, Antipov D, Gurevich AA, Dvorkin M, Kulikov AS, Lesin VM, Nikolenko SI, Pham S, Prjibelski AD. 2012 SPAdes: a new genome assembly algorithm and its applications to single-cell sequencing. J Computat Biol. 19:455–477. 10.1089/cmb.2012.0021 PMC3342519 22506599

[CIT0002] Bi G, Mao Y, Xing Q, Cao M. 2018 HomBlocks: a multiple-alignment construction pipeline for organelle phylogenomics based on locally collinear block searching. Genomics. 110:18–22. 28780378 10.1016/j.ygeno.2017.08.001

[CIT0003] Castresana J. 2002 Gblocks, v. 0.91 b. online version. [accessed 2012 February 6]; Gblocks_ server. html. http://molevol.cmima.csic.es/castresana.

[CIT0004] Hahn C, Bachmann L, Chevreux B. 2013 Reconstructing mitochondrial genomes directly from genomic next-generation sequencing reads—a baiting and iterative mapping approach. Nucleic Acids Res. 41:1–9. 23661685 10.1093/nar/gkt371 PMC3711436

[CIT0005] Katoh K, Standley DM. 2013 MAFFT multiple sequence alignment software version 7: improvements in performance and usability. Mol Biol Evol. 30:772–780. 23329690 10.1093/molbev/mst010 PMC3603318

[CIT0006] Lohse M, Drechsel O, Bock R. 2007 OrganellarGenomeDRAW (OGDRAW): a tool for the easy generation of high-quality custom graphical maps of plastid and mitochondrial genomes. Curr Genetics. 52:267–274. 10.1007/s00294-007-0161-y 17957369

[CIT0007] Lowe TM, Eddy SR. 1997 tRNAscan-SE: a program for improved detection of transfer RNA genes in genomic sequence. Nucleic Acids Res. 25:955–964. 9023104 10.1093/nar/25.5.955 PMC146525

[CIT0008] Okonechnikov K, Golosova O, Fursov M. 2012 Unipro UGENE: a unified bioinformatics toolkit. Bioinformatics. 28:1166–1167. 22368248 10.1093/bioinformatics/bts091

[CIT0009] Ronquist F, Teslenko M, van der Mark P, Ayres DL, Darling A, Höhna S, Larget B, Liu L, Suchard MA, Huelsenbeck JP, et al 2012 MrBayes 3.2: efficient Bayesian phylogenetic inference and model choice across a large model space. Syst Biol. 61:539–542. 22357727 10.1093/sysbio/sys029 PMC3329765

[CIT0010] Ruby JG, Bellare P, DeRisi JL. 2013 PRICE: software for the targeted assembly of components of (Meta) genomic sequence data. G3: Genes| Genomes| Genetics. 3:865–880. 23550143 10.1534/g3.113.005967 PMC3656733

[CIT0011] Stamatakis A. 2014 RAxML version 8: a tool for phylogenetic analysis and post-analysis of large phylogenies. Bioinformatics. 30:1312–1313. 24451623 10.1093/bioinformatics/btu033 PMC3998144

[CIT0012] Wick RR. 2015 Bandage: interactive visualization of de novo genome assemblies. Bioinformatics. 31:3350–3352. 26099265 10.1093/bioinformatics/btv383 PMC4595904

